# Establishment of a Necroptosis Related Genes Signature to Predict Prognosis and Therapeutic Response in Colon Cancer

**DOI:** 10.3389/fcell.2022.921320

**Published:** 2022-07-08

**Authors:** Yuan Wang, Yongbiao Huang, Chunya Li, Xi Wang, Mu Yang, Duo Xu, Bo Liu, Xianglin Yuan

**Affiliations:** Department of Oncology, Tongji Hospital, Tongji Medical College, Huazhong University of Science and Technology, Wuhan, China

**Keywords:** tumor microenvironment, immunotherapy, survival, necroptosis, colon cancer

## Abstract

Necroptosis, as a form of programmed cell death, is involved in many physiological and pathological processes. However, its role in cancer progression and therapeutic response remains controversial. Colon cancer is one of the leading causes of cancer death and patients’ response to immune checkpoint blockade vary to a large degree. In this study, we investigated necroptosis related genes (NRGs) alterations in colon cancer by bioinformatics analysis. Colon cancer patients were classified into two subtypes with distinct clinical and molecular features based on NRGs. After finding differentially expressed genes and lasso regression, a prognostic model based on four necroptosis signature genes was constructed. The necroptosis signature was also a good predictor in the field of chemotherapy and immunotherapy in colon cancer. Altogether, this study illustrates the relationship between necroptosis and colon cancer, and establishes a novel scoring method to predict prognosis and therapeutic response in colon cancer patients.

## Introduction

Necroptosis was first reported in 2005 as a programmed form of cell death which exhibited features of both necrosis and apoptosis (Degterev et al., 2005). Key necroptosis effector molecules involved in necroptosis were RIPK1, RIPK3, and MLKL. Receptor-interacting serine/threonine protein kinase 1 (RIPK1) was first identified as a regulator of cell death (Hsu et al., 1996). In 2008, it was identified as the target of necrostatin-1 (Nec-1), which suppressed caspase inhibition-mediated cell death (Degterev et al., 2008). RIPK3, another member of the RIPK family, was shown to be crucial for death receptor-triggered necroptosis in 2009 (Cho et al., 2009; He et al., 2009; Zhang et al., 2009). Mixed-lineage kinase domain-like protein (MLKL) was identified to participate in necroptosis after activation of RIPK3 in 2012 (Sun et al., 2012), however, published reports provided conflicting mechanisms on how it led to membrane rupture (Cai et al., 2014; Chen et al., 2014; Dondelinger et al., 2014; Hildebrand et al., 2014; Wang et al., 2014). Necroptosis can be induced by either RIPK1-dependent or RIPK1-independent mechanisms upon diverse stimuli. In RIPK1-dependent necroptosis, the binding of tumor necrosis factor (TNF) to tumor necrosis factor receptor 1 (TNFR1) induces a conformational change in TNFR1 trimers. TNFR1 subsequently leads to the recruitment of downstream proteins, including RIPK1, TRAF2 (TNFR-associated factor 2), TRAF5, TRADD (TNFR-associated death domain), cIAP1 (cellular inhibitor of apoptosis protein 1), and cIAP2. This membrane-bound protein complex is called complex I (Vandenabeele et al., 2010). A cytosolic death-inducing complex comprised of FADD (FAS-associated death domain protein), RIPK1, caspase-8 and TRADD is formed afterwards, which is known as complex II (Tenev et al., 2011). Deubiquitinated of RIPK1 switches the cell death mode from apoptosis to necroptosis (Vandenabeele et al., 2010). Autophosphorylated RIPK1 interacts with RIPK3 through their RIP homotypic interaction motif (RHIMs) (Li et al., 2012), leading to the formation of the necrosome complex (Li et al., 2012). Mitochondrial reactive oxygen species (ROS) (Zhang et al., 2017) and cylindromatosis (CYLD) (Moquin et al., 2013) was reported to be important for necrosome formation. In necrosomes, RIPK3 phosphorylates its substrate MLKL. MLKL is then oligomerized and translocated to the plasma membrane, leading to the execution of necroptosis. In RIPK1-independent necroptosis, inducers including Toll-like receptor 3 (TLR3), TLR4 and interferons (IFNs) can directly recruit and activate RIPK3 and MLKL (Weinlich et al., 2017). RIPK1 behaves in an inhibitory manner in combination with caspase 8, FADD and FLIP (FLICE-like inhibitory protein) in this case (Weinlich et al., 2017).

Necroptosis is a double-edged sword in many cancer types. On the one hand, necroptosis plays an antitumor role as a form of programmed cell death. On the other hand, necroptosis triggers inflammatory responses and is reported to promote cancer metastasis and immunosuppression (Gong et al., 2019). Colorectal cancer (CRC) is the second most common cause of cancer death in the United States (Siegel et al., 2020). In CRC, although the tumor-suppressing effects of RIPK3 and RIPK1 have been discovered (Moriwaki et al., 2015), RIPK3-mediated inflammation was reported to promote intestinal tumors by inducing an immune-suppressive tumor microenvironment (TME) (Jayakumar and Bothwell, 2019; Liu et al., 2019), and RIPK1 has been shown to interact with mitochondrial Ca^2+^ uniporter (MCU) to promote colorectal oncogenesis (Zeng et al., 2018). Besides, MLKL exhibits a suppressive effect during intestinal tumorigenesis in various researches (Zhao et al., 2019; Zhao et al., 2021). And it was reported that genetic deletion of MLKL had no impact on colon cancer development (Alvarez-Diaz et al., 2021). One possible reason for such differences might be that necroptotic cells can release various regulatory cytokines, which can either facilitate neoplastic progression by stimulating the proliferation of neighboring cells or result in tumor cell elimination by activating cytotoxic CD8^+^ T lymphocytes (Gong et al., 2019). A growing number of studies have reported the anti-tumor effect of chemotherapy (Oliver Metzig et al., 2016), radiotherapy (Nehs et al., 2011) and immunotherapy (Van Hoecke et al., 2018) in a necroptosis-dependent manner. These findings shed light on the complexity and importance of necroptosis in cancer. Therefore, it is urgent to systematically analyze the relationship between necroptosis and colon cancer progression and the therapeutic response.

**FIGURE 1:**
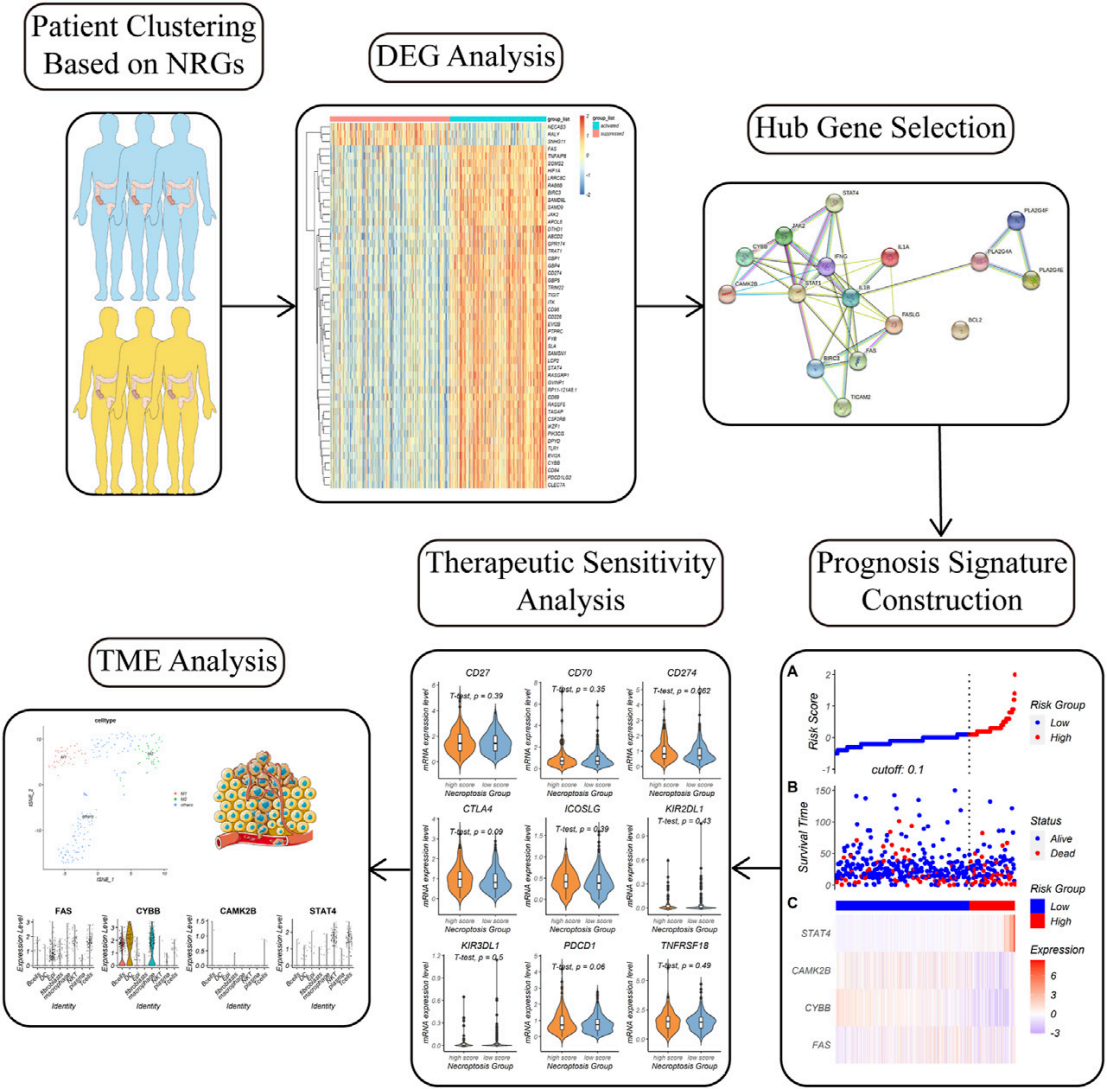
Workflow of the study. Colon cancer patients were divided into two subgroups with distinct molecular and clinicopathologic features based on necroptosis related genes (NRGs). Differential gene analysis, hub gene analysis, lasso regression, and multivariate Cox regression was carried step by step to figure out a necroptosis signature. The necroptosis signature could predict prognosis and therapeutic response in colon cancer patients. We also investigated the relationship between the necroptosis signature and tumor microenvironment (TME) by single-cell analysis.

The design of this research is in Figure 1. In this study, we downloaded genes related to necroptosis from Gene Ontology (GO) database and the Kyoto Encyclopedia of Genes and Genomes (KEGG) database to obtain a list of necroptosis related genes (NRGs). We then identified two subtypes based on the NRGs in the TCGA-COAD cohort. The two NRGs-based subtypes have distinct clinical features and molecular characteristics. After differentially expressed gene analysis and Lasso regression, a total of four necroptosis signature genes were included to establish a prognostic model. On the basis of the necroptosis scoring model, responses to chemotherapy and immunotherapy were analyzed. Single-cell analysis revealed the difference in tumor microenvironment between the two necroptosis groups. Our study shed light on the essential role of necroptosis in colon cancer, which could be useful in prognosis prediction and guiding therapy in clinical practice.

## Materials and Methods

### Study Population

The RNA transcriptome datasets (HTSeq-counts and HTSeq-FPKM) and the relevant clinical information of 469 primary colon cancer patients from the TCGA Colon Cancer (TCGA-COAD) cohort were downloaded from UCSC Xena website (https://xenabrowser.net/datapages/). Raw counts data was used for identifying differentially expressed genes. For the other analysis, Fragments Per Kilobase of transcript per Million mapped reads (FPKM) was transformed into log2 (FPKM +1). Corresponding somatic mutation profile was also downloaded from UCSC Xena website. Patients in the TCGA-COAD cohort were divided into the training set and validation set randomly. The training set was used for constructing LASSO regression model and the model was validated in the validation set.

Two datasets, GSE28722 and GSE17538, were downloaded using the R package “GEOquery.” GSE28722 and GSE17538 contain mRNA and survival profiles of 129 CRC patients and 244 colon cancer patients respectively.

Single-cell transcriptome file and clinical data of 12 CRC samples of GSE166555 was downloaded from the Gene expression omnibus (GEO) database (https://www.ncbi.nlm.nih.gov/geo/).

Patient phenotype information was listed in Supplementary Table S1. Baseline characteristics including age, gender, and tumor stage among the four datasets were similar, and also fitted well with previous studies (Siegel et al., 2020).

### Acquisition of Necroptosis Related Genes

The necroptosis gene set “hsa04217” contains 159 NRGs and was downloaded from the KEGG database by using the R package “KEGGREST.” Another necroptosis gene set “0097528” containing three NRGs was downloaded from the GO database using the R package “GO.db.”

### Necroptosis Related Genes Based Clustering

We performed K-means consensus clustering with the FPKM matrix of 159 NRGs to identify subgroups in the TCGA-COAD cohort. Consensus clustering was carried out using the function “ExecuteCC” in the R package “CancerSubtypes” (Xu et al., 2017). The number of clusters was determined based on both the clustering results and clinical significance. Heatmap was generated by using the function “drawHeatmap” in the R package “CancerSubtypes”. Data were normalized using “max_min” [(value−min)/(max−min)] before drawing the heatmap.

### Clinical and Mutational Characteristics of Necroptosis Subgroups

We then compared clinicopathological and molecular characteristics between the two necroptosis subgroups previously identified. Clinical information including pathological and clinical stage were extracted for comparison and the results were presented in bar charts. The Kaplan-Meier (K-M) method was performed to compare overall survival (OS) between the two necroptosis subgroups. The somatic mutation data were further analyzed using the R package “maftools” (Mayakonda et al., 2018). Somatic mutation types that “maftools” could detect include synonymous variant, missense variant, stop-gain, frameshift variant, three prime untranslated region (3′-UTR) variant, intron variant, and multi-hit. It can also provide the concrete basepair substitution information in each individual.

### Immune Cell Infiltration Analysis of Necroptosis Subgroups

To examine the relationship between necroptosis and the immune microenvironment, “CIBERSORT” was used to compare the absolute abundance of 22 human hematopoietic cell phenotypes between the two necroptosis subgroups. CIBERSORT is a tool for deconvolution of the expression matrix of human cell subtypes from tissue gene expression profiles based on the principle of linear support vector regression (Newman et al., 2015). Cell types CIBERSORT could identify include seven T-cell subsets, naïve and memory B cells, natural killer (NK) cells, plasma cells and myeloid subtypes. The standard annotation file LM22 containing 547 genes was provided as input.

### Differentially Expressed Genes Identification

Differentially expressed genes (DEGs) between the NRGs-activated and NRGs-suppressed subtypes were identified by using the R package “limma.” NRGs with | log2 (fold change) | > 1 and adjusted *p* value <0.05 were considered as necroptosis subtype specific genes. Gene set enrichment analysis (GSEA) method was carried out to determine the signaling pathways the DEGs involved in with the R package “clusterProfiler.” For GSEA analysis using the function “gseKEGG,” permutation number was set at 1,000, minimal size of each gene set for analyzing was set at 120, *p* cutoff value was set at 0.9, and “BH” was chosen for the “pAdjustMethod” parameter. Adjusted *p* value was set at 0.05 for figuring out significantly up-regulated and downregulated pathways.

### Hub Genes Selection

Proteins are executors of biological processes. To extend our research conclusion from genomics to proteomics, we generated a protein-protein interaction (PPI) network including both functional and physical associations by importing the DEGs related to necroptosis into STRING (https://www.string-db.org/) (Szklarczyk et al., 2021). The false discovery rate (FDR) was set at 0.05 and the minimum interaction score was set at 0.4. Then we processed the result with the Cytoscape software (version 3.9.0). Hub genes were identified by the Degree algorithm using the “cytoHubba” plugin (Chin et al., 2014).

### Establishment and Validation of a Prognostic Necroptosis Signature

To determine significant prognostic genes among the hub genes, we then applied the Least absolute shrinkage and selection operator (LASSO) method for variable selection in a Cox regression model by using the R packages “lars” and “glmnet.” We extracted the hub genes related to necroptosis when the first-rank value of Log(*λ*) was the minimum likelihood of deviance. A multivariate Cox regression analysis was then used to investigate the correlation between the expression levels of the necroptosis signatures and the overall survival (OS) of patients in the TCGA-COAD cohort using the R package “survival.” The necroptosis score could be calculated based on the Cox model using the formula:

$$\text{Necroptosis Score}=\text{exp}\left[\overset{p}{\underset{i=1}{\sum }}b_{i}X_{i}-\overset{p}{\underset{i=1}{\sum }}b_{i}{\bar{X}}_{i}\right]$$

• The coefficients (b_1_, b_2_, … , b_p_) are the coefficients of each gene in the Cox model.
• Xi is the mRNA expression level of the ith gene.
• ${\bar{X}}_{i}$ is the mean mRNA expression level of the ith gene.

Based on the necroptosis score, patients in the TCGA-COAD cohort, GSE28722, and GSE17538 datasets were divided into high-necroptosis score and low-necroptosis score subgroups. The function “ComBat” in the R package “sva” (Leek et al., 2012) was used to remove batch effects from the GSE28722 and GSE17538 datasets. The optimal cutoff point for necroptosis score was calculated using the R package “survminer” according to the expression level and the survival information. The K-M method was performed to compare overall survival (OS) between the two subgroups in the GSE28722 and GSE17538 datasets respectively.

### Construction and Validation of Nomogram Based on the Necroptosis Signature

With R package “rms,” the necroptosis score, age, gender, and tumor stage of the colon cancer patients in the TCGA-COAD cohort were used to set up a nomogram for the 1-, 2-, and 5-year OS. Calibration curves were generated to evaluate the agreement between the actual and predicted survival probabilities at 1-, 2-, and 5-year. Parameters m and B in the function “calibrate” were both set at 100. The 1-, 3-, and 5-year time-dependent receiver operating characteristics (ROC) curves of the model were generated by the R package “survivalROC” (Heagerty and Zheng, 2005).

### Prediction of Necroptosis Signature in the Field of Chemotherapy and Immunotherapy

We applied the R package “oncoPredict” to predict clinical response to multiple chemotherapy drugs in the high-necroptosis score and low-necroptosis score groups, which is based on Genomics of Drug Sensitivity in Cancer (GDSC) and Cancer Therapeutics Response Portal (CTRP) (Maeser et al., 2021).

The immunotherapy response prediction of the two subgroups was estimated with tumor immune dysfunction and exclusion (TIDE) score and tumor inflammation signature (TIS) score. TIDE is a computational method that could be used to predict immune checkpoint blockade (ICB) response in cancer patients by computing T cell dysfunction and T cell exclusion (Jiang et al., 2018). We obtained TIDE scores and T cell dysfunction scores from the TIDE web (http://tide.dfci.harvard.edu/). TIS score was calculated as an average value of log2-scale normalized expression of 18 signature genes to predict clinical response to PD-1 blockade (Ayers et al., 2017). Besides, we compared mRNA expression levels of commonly accepted immunotherapy-related genes between the high-necroptosis score and low-necroptosis score subgroups.

### Single-Cell Analysis for Necroptosis Heterogeneity Estimation

After filtering out low low-quality cells, single-cell transcriptomic data of cells from 12 primary samples in GSE166555 was used for further analysis (Uhlitz et al., 2021). Cell clusters were annotated based on previously reported cell type-specific signatures and marker genes. R package “Seurat” was used to process the data and generate t-SNE plot for cell types visualization. The function “AddModuleScore” was applied to calculate score of the necroptosis signature. The functions “FindNeighbors” and “FindClusters” were used to identify cell clusters of macrophages.

### Statistical Analysis

For the comparison of continuous variables, the unpaired Student’s *t*-test was applied for normally distributed data, and the Wilcoxon test or Kruskal–Wallis test was performed for non-normally distributed data. Two-sided Fisher’s exact test was used to measure categorical variables between two groups. *p* < 0.05 was set as a significant difference in all statistical methods, and all *p* values were two-tailed. R software (version 4.1.1) (http://www.R-project.org) was used for data analysis and generation of figures.

## Results

### Necroptosis Related Genes Define Subgroups With Different Clinical Characteristics in Colon Cancer Patients

**FIGURE 2:**
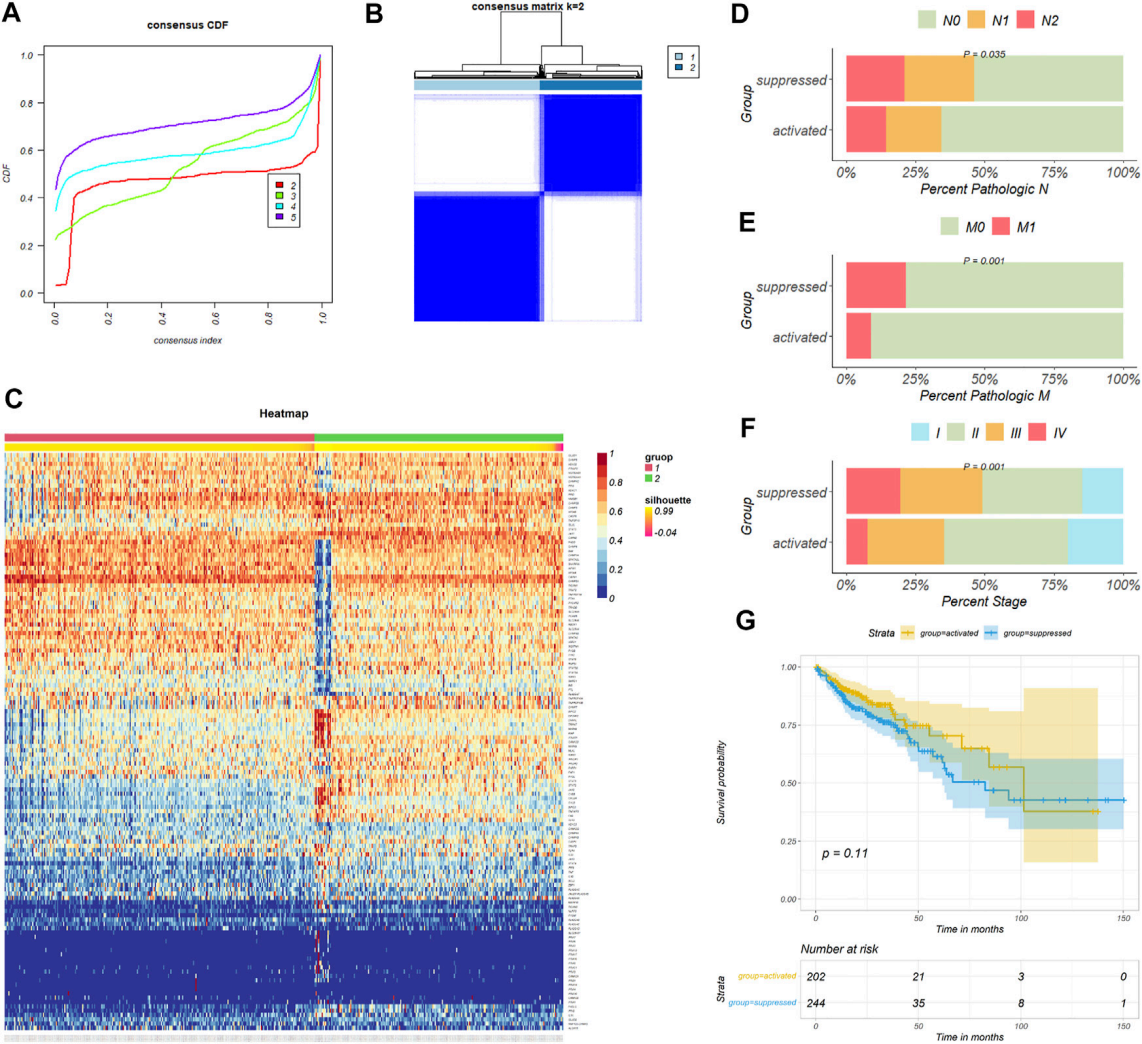
Consensus clustering for necroptosis related genes (NRGs) in colon cancer patients. **(A)** The relative change in area under the CDF curve of K = 2–5. **(B)** The consensus matrix shows patients with two heterogenous necroptosis states in the TCGA-COAD cohort. **(C)** The heatmap shows expression levels of NRGs in the NRGs-activated and NRGs-suppressed group. **(D–F)** Difference of tumor pathological N, M, and clinical stage distribution between the two necroptosis subgroups respectively. **(G)** Kaplan-Meier curves for overall survival based on necroptosis subgroups (Log-rank test) in the TCGA-COAD cohort.

159 necroptosis related genes (NRGs) involved in the pathway “hsa04217” were downloaded from KEGG database, and three NRGs were downloaded from GO database by the accession number “0097528.” After removing duplicate genes, a total of 159 NRGs were finally engaged in this study (Supplementary Table S2). 469 colon cancer patients from the TCGA-COAD cohort were then divided into distinct subtypes based on 159 NRGs expression profiles. Taking the consensus clustering results and clinical significance into consider (Figure 2A), two necroptosis subgroups were identified. Cluster 1 (*n* = 260, 55.4% of all colon cancer patients) was defined as the NRGs-suppressed subtype (Figure 2B), according to the relative downregulation of most NRGs in this cluster (Figure 2C). Cluster 2 (*n* = 209, 44.6% of all colon cancer patients) was therefore defined as the NRGs-activated subtype based on the relative upregulation of NRGs. The two subtypes showed thoroughly heterogenous clinical outcomes (Table 1). Patients in the NRGs-activated subgroup had lower pathological N stage (*p* = 0.035) (Figure 2D), pathological M stage (*p* = 0.001) (Figure 2E), and clinical stage (*p* = 0.001) (Figure 2F) compared with patients in the NRGs-suppressed subgroup. K-M plots suggested that patients who were divided into the NRGs-activated subgroup tended to have better OS relative to patients in the NRGs-suppressed subgroup, but the results might be biased by limited sample size (Figure 2G). Together, these results suggested that our clustering method based on NRGs was reasonable and had clinical significance in colon cancer patients.

**TABLE 1:** Clinical characteristics of colon cancer patients in the NRGs-activated and NRGs-suppressed group.

	NRGs-activated group	NRGs-suppressed group	*p* value	Test
Pathologic T stage (%)			0.763	Exact
T1	5 (2.4)	6 (2.3)		
T2	39 (18.7)	41 (15.9)		
T3	137 (65.6)	180 (69.8)		
T4	27 (12.9)	31 (12.0)		
Tis	1 (0.5)	0 (0.0)		
Total	209	258		
Pathologic N stage (%)			0.035	Exact
N0	137 (65.6)	139 (53.9)		
N1	42 (20.1)	65 (25.2)		
N2	30 (14.4)	54 (20.9)		
Total	209	258		
Pathologic M stage (%)			0.001	Exact
M1	16 (8.9)	49 (21.5)		
M2	164 (91.1)	179 (78.5)		
Total	180	228		
Clinical stage (%)			0.001	Exact
I	41 (19.9)	37 (14.8)		
II	92 (44.7)	90 (36.0)		
III	57 (27.7)	74 (29.6)		
IV	16 (7.8)	49 (19.6)		
Total	206	250		

### Necroptosis Related Genes-Based Subtypes Show Different Mutational and Immunological Characteristics

**FIGURE 3:**
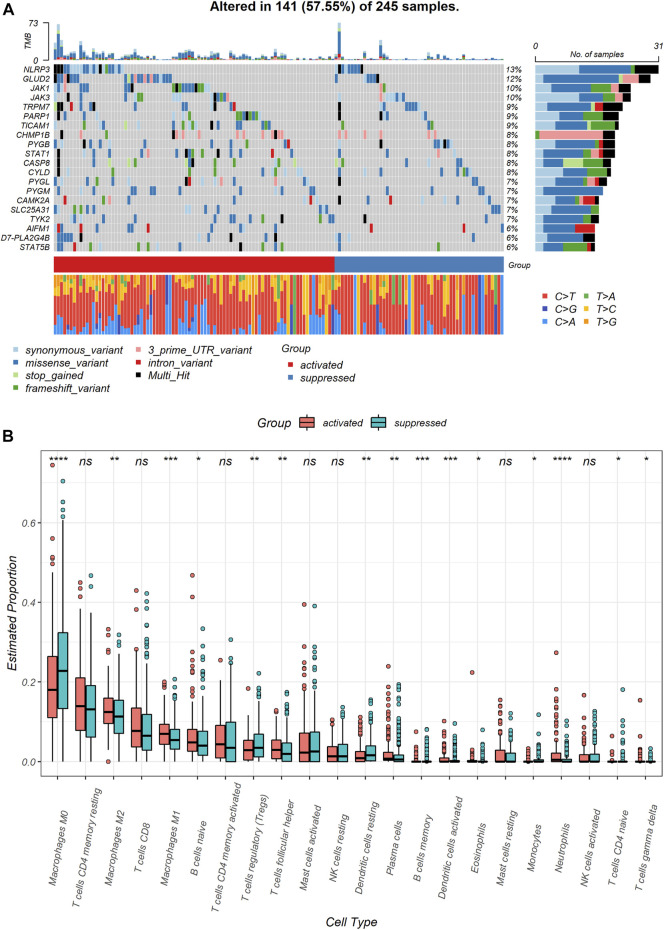
Genomic and immune profile alterations between the NRGs-activated and NRGs-suppressed subgroup. **(A)** Landscape of mutation profiles in colon cancer samples. Mutation information of each gene in each sample is shown in the waterfall plot. Top panel shows individual tumor mutation burden. **(B)** The comparison of infiltration levels of 22 immune cells between the NRGs-activated and NRGs-suppressed groups. * represents *p* < 0.05, ** represents *p* < 0.01, *** represents *p* < 0.01, **** represents *p* < 0.0001, ns represents no significant difference.

To identify genomic alterations difference between the two subgroups, we compared NRGs mutation between the NRGs-activated subgroup and the NRGs-suppressed subgroup. The most frequently mutated NRGs in the NRGs-activated subgroup and the NRGs-suppressed subgroup were NLRP3 and GLUD2 (Figure 3A). Besides, the NRGs-activated subgroup had a higher probability of necroptosis-related genes mutation than the NRGs-suppressed subgroup (Figure 3A).

Immune cell infiltration markedly influenced tumor microenvironment. Therefore, we also explored differences in immune cell infiltrations between two necroptosis subgroups (Supplementary Table S3). Notably, M0 macrophages (*p* < 0.0001) and resting dendritic cells (*p* < 0.01) were significantly downregulated in the NRGs-activated subgroup. Neutrophils (*p* < 0.0001), M1 macrophages (*p* < 0.001), activated dendritic cells (*p* < 0.001), M2 macrophages (*p* < 0.01), follicular helper T cells (*p* < 0.01), and plasma cells (*p* < 0.01) were significantly up-regulated in the NRGs-activated subgroup (Figure 3B).

### Necroptosis Subtype Signature is a Prognostic Indicator for Colon Cancer Patients

**FIGURE 4:**
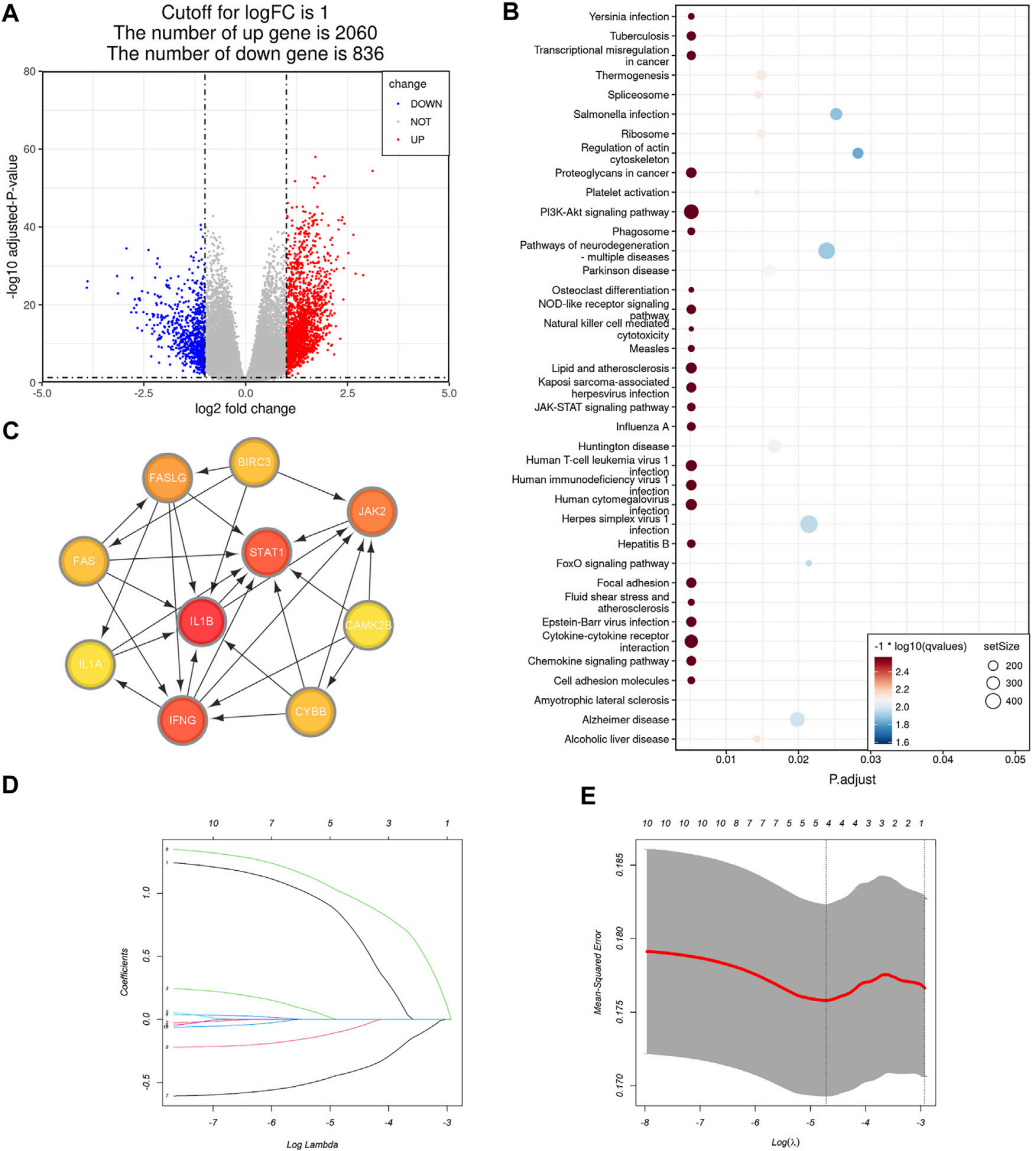
Selection of the necroptosis gene signature in colon cancer. **(A)** Volcano plot of differentially expressed genes (DEGs) between the NRGs-activated group and NRGs-suppressed group. The red dots represent upregulated genes, while the blue dots represent downregulated genes [| log2 (fold change) | > 1 and adjusted *p* value <0.05]. **(B)** Bubble plot of GSEA pathway enrichment results of the DEGs between the NRGs-activated group and NRGs-suppressed group. Size of the circle represents the size of the gene set and color of the circle is based on -log10 (q value). **(C)** Protein-protein interaction (PPI) network including both functional and physical associations of hub necroptosis genes identified in Cytoscape. Color shade of the gene correlates with its score obtained by Degree method using the “cytoHubba” plugin. **(D)** The 10-fold cross-validation for variable selection in the LASSO model. **(E)** The LASSO coefficient profile of the four necroptosis signature genes.

To obtain a more practical signature that could be used for necroptosis subtype identification, we next figured out hub DEGs between the NRGs-activated group and the NRGs-suppressed group. Differential gene analysis revealed that 2,060 genes were significantly upregulated in the NRGs-activated subgroup (Figure 4A), and 836 genes were significantly downregulated (Figure 4A; Supplementary Table S4). GSEA results revealed that the DEGs were mainly enriched in the pathways including cytokine-cytokine receptor interaction, chemokine signaling pathway, JAK-STAT signaling pathway, PI3K-Akt signaling pathway, transcriptional misregulation in caner, proteoglycans in cancer, phagosome, NOD-like receptor signaling pathway, natural killer mediated cytotoxicity, focal adhesion, and cell adhesion molecules (Figure 4B; Supplementary Table S5). Among the 2,896 DEGs, we further chose 16 NRGs to generate a PPI network in STRING. Differentially expressed necroptosis genes between the NRGs-activated and suppressed groups included JAK2, STAT4, BIRC3, CYBB, FAS, IFNG, STAT1, BCL2, FASLG, TICAM2, IL1B, PLA2G4F, PLA2G4E, PLA2G4A, IL1A, and CAMK2B. After importing the PPI network including both functional and physical associations generated by STRING into Cytoscape, we figured out 10 hub genes: IL1B, IFNG, STAT1, JAK2, FASLG, FAS, BIRC3, CYBB, CAMK2B, and STAT4 (Figure 4C). To further reduce the dimension of the necroptosis signature, we randomly allocated the patients in the TCGA-COAD cohort into the training set (*n* = 233, 53.8% of all colon cancer patients) and the validation set (*n* = 200, 46.2% of all colon cancer patients), and applied the LASSO Cox regression model to find out the most powerful prognostic necroptosis genes in the training set. This resulted in a necroptosis signature of four genes: FAS, CYBB, CAMK2B, and STAT4 (Figures 4D,E **)**.

**FIGURE 5:**
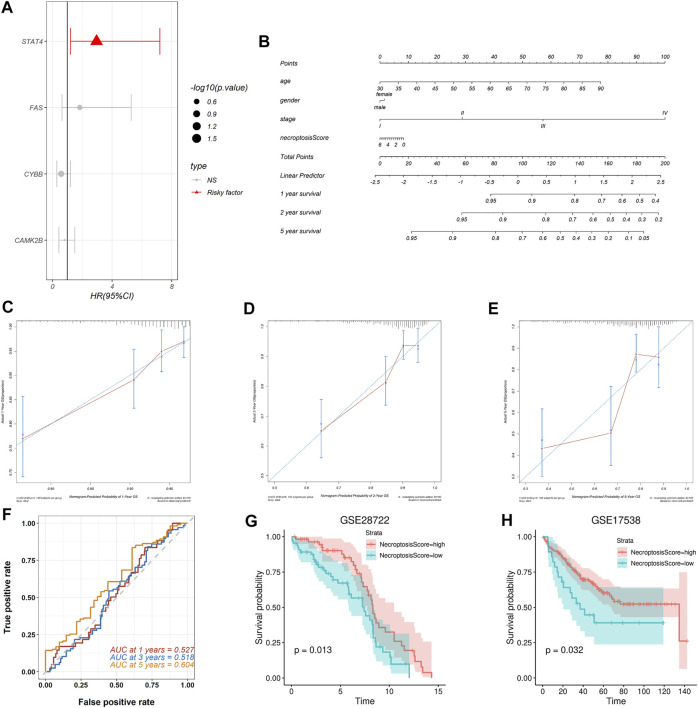
Construction and validation of the necroptosis prognosis model based on the necroptosis gene signature. **(A)** The forest plot shows multi-Cox analysis result between the four necroptosis signature genes and OS. **(B)** The nomogram integrates age, gender, tumor stage, and necroptosis score to predict the probability of the 1-, 2-, and 5-year OS. **(C,D)** The calibration curves for 1-, 2-, and 5-year OS, respectively. **(E)** The 1-, 3-, and 5-year time-dependent ROC for necroptosis signature survival prediction in the TCGA-COAD database, respectively. **(F–H)** Validation of the necroptosis signature in predicting the prognosis of patients in GSE28722 and GSE17538 datasets respectively.

A multivariate Cox proportional hazards model was used to find the relationship between the necroptosis signature genes and OS in the TCGA-COAD cohort and the results were displayed in a forest plot (Figure 5A). Among all the necroptosis signature genes, the oncogene signal transducer and activator of transcription 4 (STAT4) is a strong predictor of OS in colon cancer patients (*p* = 0.01688, HR = 2.9599, 95%CI: 1.2153–7.209) (Figure 5A). Necroptosis scores of each patient in the TCGA-COAD cohort were calculated based on the Cox model as previously mentioned. A nomogram was constructed combining age, gender, and tumor stage with necroptosis score to offer clinicians a quantitative approach for predicting OS in colon cancer patients (Figure 5B). A higher necroptosis signature score is correlated with worse overall survival (Figure 5B). The calibration curve showed good concordance between the observations and the predictions at 1-, 2-, and 5-year (Figures 5C–E). In addition, time-dependent receiver operating characteristics (ROC) were used to evaluate the sensitivity and specificity of the prognostic model based on the necroptosis signature. The 1-, 3-, and 5-year AUC of the TCGA-COAD cohort were 0.527, 0.518, and 0.604, respectively (Figure 5F). Together, these results indicated that the necroptosis signature could be used for prognosis prediction in colon cancer patients.

To validate prognostic predictive power of the necroptosis signature, we assessed its performance in two independent GEO cohorts. We calculated the necroptosis score for each patient and separated the patients in each cohort into the high-necroptosis score group and low-necroptosis score group as previously mentioned. Kaplan-Meier survival analysis revealed that patients in the high-necroptosis score group had better OS than the low-necroptosis score group, both in the dataset GSE28722 (*p* = 0.013) (Figure 5G) and the dataset GSE17538 (*p* = 0.032) (Figure 5H). These results suggested that the necroptosis signature we proposed could be extended to other colon cancer cohorts to predict prognosis.

### Necroptosis Subtype Signature is a Predictor for Chemotherapy and Immunotherapy

**FIGURE 6:**
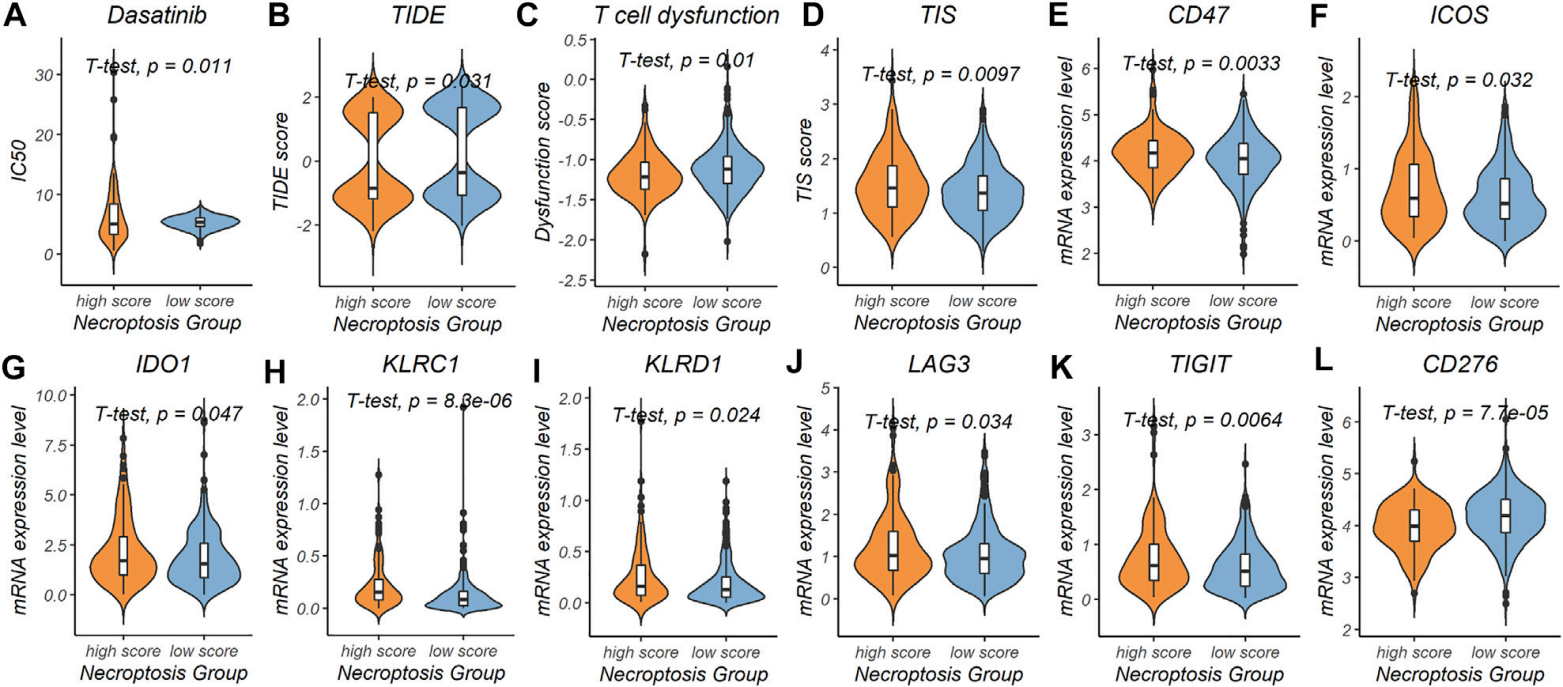
Predictive power of the necroptosis gene signature in chemotherapy and immunotherapy response. **(A)** Prediction of IC50 for dasatinib in the high-necroptosis score and low-necroptosis score group. **(B–D)** Comparison of tumor immune dysfunction and exclusion (TIDE) score, T cell dysfunction score and tumor inflammation signature (TIS) score between the high-necroptosis score and low-necroptosis score group. **(E-L)** mRNA expression level differences of immunotherapy related genes (CD47, ICOS, IDO1, KLRC1, KLRD1, LAG3, TIGIT, and CD276) between the high-necroptosis score and low-necroptosis score group.

To explore the prediction power of the necroptosis subtype signature in the field of chemotherapy and immunotherapy response, patients in the TCGA-COAD cohort were divided into the high-risk subgroup (*n* = 122, 28.2% of all colon cancer patients) and the low-risk subgroup (*n* = 311, 71.8% of all colon cancer patients) based on the necroptosis risk score. After evaluating 198 chemotherapy drugs (Supplementary Table S6), the low-necroptosis score subgroup was found to have lower half-maximal inhibitory concentration (IC50) for dasatinib compared with the high-necroptosis score subgroup (*p* = 0.011) (Figure 6A). This indicated that colon cancer patients with low necroptosis score might benefit from dasatinib.

We further used the TIDE score and the T cell dysfunction score to evaluate possible response to immunotherapy in the high- and low-necroptosis score subgroup (Supplementary Table S7). Patients in the high-necroptosis score subgroup were characterized by a significantly lower TIDE score (*p* = 1.9e-14) and T cell dysfunction score (*p* = 1.9e-14), and higher TIS score (*p* = 0.0097) compared with patients in the low-necroptosis score subgroup (Figures 6B–D). This indicated that high-necroptosis score patients seemed to be more sensitive to ICB. We also compared mRNA expression levels of 17 previously reported immunotherapy related genes between the high- and low-necroptosis score subgroup (Supplementary Table S2). Interestingly, distinct necroptosis score groups had different immunotherapy-related genes expression levels. CD47 (*p* = 0.0033), ICOS (*p* = 0.032), IDO1 (*p* = 0.047), KLRC1 (*p* = 8.3e-06), KLRD1 (*p* = 0.024), LAG3 (*p* = 0.034), and TIGIT (*p* = 0.0064) were up-regulated in the high-necroptosis score subgroup (Figures 6E–K), while CD276 was up-regulated in the high-necroptosis score subgroup (*p* = 7.7e-05) (Figure 6L). Together, these results revealed that the necroptosis signature we proposed was also helpful in predicting therapeutic response in colon cancer patients.

### Necroptosis Signature is Heterogeneous in Tumor Immune Microenvironment

**FIGURE 7:**
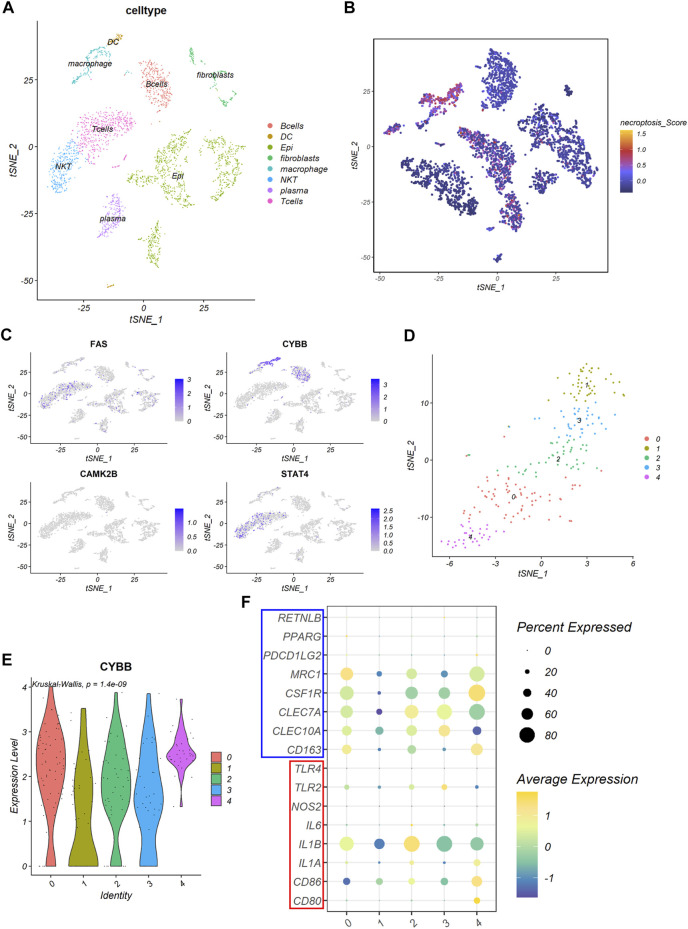
The expression of the necroptosis signature in colon cancer. **(A)** The t-SNE plot shows percentage of eight cell types in colon cancer tissues. **(B)** The t-SNE plot shows necroptosis score of each cell cluster. **(C)** The t-SNE plots shows expression levels of FAS, CYBB, CAMK2B, and STAT4 in each cell cluster. **(D)** The t-SNE plot shows five clusters of macrophages, denoted by 0–4, identified by the R package Seurat. **(E)** The violin plot shows expression levels of CYBB among the five macrophage clusters. **(F)** The bubble plot shows expression of macrophage marker genes among the five macrophage clusters. Gens in the red box are M1 macrophage marker genes and genes in the blue box are M2 macrophage marker genes.

As previous GSEA result showed that DEGs between the NRGs-activated group and the NRGs-suppressed group were enriched in proteoglycans in cancer, focal adhesion, cell adhesion molecules, and natural killer mediated cytotoxicity, we further explore the relationship between necroptosis and TME at a single-cell level. Based on the previously reported cell type-specific signatures and marker genes (Supplementary Table S2), cells from 12 primary CRC patients were divided into eight types, including B cell, dendritic cell, epithelial cell, fibroblast, macrophage, NKT cell, plasma cell, and T cell (Figure 7A). Results from single-cell analysis revealed that expression levels of four necroptosis signature genes varied to a large degree in different cell clusters. The necroptosis signature genes were significant upregulated in some particular cell types, especially macrophages (Figure 7B). Among all the necroptosis signatures, CYBB was the most frequently upregulated necroptosis signature in macrophages (Figure 7C).

Based on this result, we picked the macrophages cluster out for further exploration. Five clusters were identified using the R package “Seurat” according to gene expression level (Figure 7D). We found that the expression of CYBB were quite different among different clusters of macrophages (*p* = 1.4e-09), with cluster 0 and cluster 4 had the highest expression level among all the macrophage clusters (Figure 7E). Further analysis revealed that cluster 0 expressed a high level of MRC1, while cluster 4 expressed a high level of CSF1R (Figure 7F). This indicated that cluster 0 and cluster 4 all exhibited molecular characteristics of M2 macrophages.

## Discussion

Necroptosis is a form of programmed cell death which has attracted more and more attention in recent years. However, conflicting evidence existed in studies about the relationship between necroptosis and different tumor types. Inducing tumor cell necroptosis is a potential treatment strategy but might be a double-edged sword. Colorectal cancer (CRC) is the second most common cause of cancer death in the United States. Therefore, analyses of the relationship between necroptosis and colon cancer were performed to attain a better understanding of the mechanisms of how necroptosis involved in oncogenesis, development, and metastasis of colon cancer. Immunotherapy is proving to be an effective therapeutic approach in a variety of cancers. But only a subset of cancer patients exhibits durable responses, suggesting that a deeper investigation of cancer immunity is required. Thus, we also explored whether different levels of NRGs expression indicated suitable targets for different therapies in colon cancer.

First, we revealed that colon cancer patients could be characterized into two distinct necroptosis statuses with different characteristics. Patients in the NRGs-activated subgroup were characterized by aggressive clinical behavior, such as advanced pathological N, M, and clinical stage. However, no difference in the pathological T stage between the two subgroups was discovered. This indicated that necroptosis might play an important role in the metastasis in colon cancer development. We also identified the mutational characteristics of distinct necroptosis subtypes in colon cancer patients. We found that NLRP3 and GLUD2 were the most common necroptosis-related gene alteration in colon cancer patients. Glutamate dehydrogenase 2 (GLUD2) overexpression was found to inhibit glioblastoma cell growth (Franceschi et al., 2018). Our findings provided novel potential drug targets for the control of colon cancer progression and metastasis. Besides, the NRGs-activated subtype had more somatic mutations of NRGs, suggesting that NRGs mutation burden may predict the clinical and pathological characteristics of colon cancer patients.

Next, we proposed a four gene necroptosis signature for the prediction of prognosis and therapeutic response in colon cancer patients. DEGs between the NRGs-activated subgroup and NRGs-suppressed subgroup were mainly enriched in tumor-related signaling pathways including JAK-STAT signaling pathway and PI3K-Akt signaling pathway, transcriptional misregulation and proteoglycans in cancer. Besides, pathways relating to the regulation of the TME, such as focal adhesion, cell adhesion molecules, and natural killer mediated cytotoxicity, were also involved. It has been reported that chlorpyrifos could induce necroptosis in fish liver cells by regulating the ROS/PTEN/PI3K/AKT axis (Wang et al., 2020). Proteoglycans consist a large proportion of the extracellular matrix (ECM) (Theocharis and Karamanos, 2019), and dysregulation of ECM dynamics leads to the development of cancer (Walker et al., 2018). Cell adhesion contributed a lot to cancer metastasis (Khalili and Ahmad, 2015), and focal adhesion kinase (FAK) was recognized as an anti-cancer target (Dawson et al., 2021). A previous study found that inhibition of cell-surface proteins induced by disintegrin and metalloproteases (ADAMs) disrupted cell adhesion while accelerating necroptosis (Cai et al., 2016). More studies are needed to explore the crosstalk between necroptosis and aforementioned pathways. For chemotherapy, our finding suggested that colon cancer patients with low necroptosis signature score might benefit from the selective tyrosine kinase receptor inhibitor dasatinib. Dasatinib plays an antitumor role in a variety of tumor types by triggering apoptosis of tumoral cells and changing tumor microenvironment (Montero et al., 2011). Our finding favored the combination use of dasatinib with other drugs to obtain a synergistic effect in certain colon cancer patients.

Our results also emphasized the important role of the necroptotic process in cancer immunity. The NRGs-activated subgroup and the NRGs-suppressed subgroup had significantly different tumor microenvironment. Activation of NRGs seemed to be related with inflammation by activating neutrophils, macrophages, dendritic cells, T cells, and B cells. Tumor-associated neutrophils (TANs) can play either pro- or anti-tumor roles depending on the subtypes they are polarized to upon external cues (Powell and Huttenlocher, 2016). Proportion of TANs subtypes needs further exploration to understand the relationship between necroptosis and TANs in the tumor microenvironment. Single-cell analysis highlighted the important relationship between necroptosis and macrophages in colon cancer, and also figured out the possible target turning cold tumors into hot tumors. Tumor-associated macrophages (TAMs) can be polarized toward either a pro-inflammatory (M1) state or an anti-inflammatory (M2) state upon various stimulation (Vitale et al., 2019). CYBB is a subunit of the NADPH oxidase complex 2 (NOX2). NOX2 and IL1B are all important pro-inflammation factors of phagocytes. Our research showed that CYBB (Cytochrome B-245 Beta Chain) was the most apparently upregulated necroptosis signature genes in macrophages, especially in M2 macrophages. This highlighted a novel target on macrophages to remodel TME. However, the underlying mechanism between NRGs and tumor immunity is still poorly understood and warrants further investigation.

In the field of immunotherapy, microsatellite instability-high (MSI-H)/deficient mismatch repair (dMMR) was first identified as a predictive biomarker of PD-1 blockade in a clinical trial (Tabernero et al., 2015). Anti-PD-1 antibody was only effective in dMMR patients (Tabernero et al., 2015). However, the proportion of patients with microsatellite stable (MSS) and proficient MMR (pMMR) metastatic colorectal cancer (mCRC) consists of more than 95% of mCRC patients (Yaeger et al., 2018), which means that the majority of CRC patients could not benefit from immunotherapy. We discovered that patients with high-necroptosis score were potential responders to anti-PD1 and anti-CTLA4 therapy. This discovery provided theoretical support to the combination use of necroptosis inducers and immunotherapy to achieve a better therapeutic response in cancer patients. Physically induced necroptosis had already been proved to enhance the antitumor response of immune checkpoint blockade therapy (Um et al., 2020). Polyinosinic-polycytidylic acid (PolyI:C), a member of TLR family, is a necroptosis-inducing agent in tumor cells (Takemura et al., 2015). It was reported that PolyI:C could enhance the therapeutic effects of cancer immunotherapy by promoting T cell infiltration (Sultan et al., 2020). However, low-necroptosis score subgroup could benefit from targeting certain molecules, like CD276 on CD8^+^ T cells. This finding suggested that immunotherapeutic treatment should be customized according to the necroptosis state of colon cancer patients. Our discovery might serve as a useful tool to identify colon cancer patients who might potentially benefit most from precision immunotherapy.

However, as the conclusions of this study were based on bioinformatic analysis using the TCGA and GEO databases, the relationship between necroptosis and colon cancer clinical characteristics needs further validation in prospective studies. The mechanisms underlying the effects of the necroptosis signature genes on colon cancer TME also needs to be verified experimentally.

In conclusion, this study revealed the significant relationship between necroptosis and colon cancer based on bioinformatics analysis. Identifying specific necroptosis state could help in colon cancer clinical management and decision-making process. The necroptosis signature we established could help in predicting prognosis among colon cancer patients and assists in developing more effective therapeutic targets in colon cancer.

## Data Availability

Data generated and analyzed in the current study are available from the UCSC TCGA data portal (http://xena.ucsc.edu/public/) and GEO databases (dataset ID: GSE28722, GSE17538 and GSE166555, https://www.ncbi.nlm.nih.gov/).

## Supplementary Material

Supplementary Table S1 Patient phenotype information of the TCGA-COAD, GSE28722, GSE17538, and GSE166555 datasets.

Supplementary Table S2 Necroptosis related genes, immunotherapy related genes and cell marker genes used in this article.

Supplementary Table S3 CIBERSORT analysis result in the TCGA-COAD cohort.

Supplementary Table S4 Differentially expressed genes between the NRGs-activated group and the NRGs-suppressed group.

Supplementary Table S5 Results of GSEA of DEGs between the NRGs-activated group and the NRGs-suppressed group.

Supplementary Table S6 OncoPredict result of the high-necroptosis score subgroup (*n* = 122, 28.2% of all colon cancer patients) and the low-necroptosis score subgroup (*n* = 311, 71.8% of all colon cancer patients) in the TCGA-COAD cohort.

Supplementary Table S7 TIDE result of the high-necroptosis score subgroup (*n* = 122, 28.2% of all colon cancer patients) and the low-necroptosis score subgroup (*n* = 311, 71.8% of all colon cancer patients) in the TCGA-COAD cohort.
